# A screening assay for Selective Dimerizing Glucocorticoid Receptor Agonists and Modulators (SEDIGRAM) that are effective against acute inflammation

**DOI:** 10.1038/s41598-018-31150-w

**Published:** 2018-08-27

**Authors:** Jolien Souffriau, Melanie Eggermont, Sara Van Ryckeghem, Kelly Van Looveren, Lise Van Wyngene, Evelien Van Hamme, Marnik Vuylsteke, Rudi Beyaert, Karolien De Bosscher, Claude Libert

**Affiliations:** 1 Center for Inflammation Research, VIB, Ghent, Belgium; 2 Department of Biomedical Molecular Biology, Ghent University, Ghent, Belgium; 3 Bio Imaging Core, Center for Inflammation Research, VIB, Ghent, Belgium; 4 GNOMIXX ltd, Statistics for Genomics, Melle, Belgium; 5 Receptor Research Laboratories, Nuclear Receptor Lab, Center for Medical Biotechnology Center, VIB, Ghent, Belgium; 6 Department of Biochemistry, Ghent University, Ghent, Belgium

## Abstract

It has been suggested that glucocorticoid receptor (GR) agonists that promote GR homodimerization more than standard glucocorticoids such as Dexamethasone could be more effective anti-inflammatory molecules against acute and life-threatening inflammatory conditions. To test this hypothesis, we set up a screening pipeline aimed at discovering such Selective Dimerizing GR Agonists and Modulators (SEDIGRAM). The pipeline consists of a reporter gene assay based on a palindromic glucocorticoid responsive element (GRE). This assay represents GR dimerization in human A549 lung epithelial cells. In the pipeline, this is followed by analysis of endogenous GRE-driven gene expression, a FRET assay confirming dimerization, and monitoring of *in vitro* and *in vivo* anti-inflammatory activity. In a proof of principle experiment, starting from seven candidate compounds, we identified two potentially interesting compounds (Cortivazol and AZD2906) that confer strong protection in a mouse model of aggressive TNF-induced lethal inflammation. A screening pipeline for SEDIGRAM may assist the search for compounds that promote GR dimerization and limit overwhelming acute inflammatory responses.

## Introduction

Worldwide, hundreds of millions of people suffer from inflammatory diseases such as rheumatoid arthritis, psoriasis, inflammatory bowel disease and asthma. With the expanding world population, the increase in life expectancy and the relative increase in auto-immune and auto-inflammatory diseases, the market of anti-inflammatory therapies has been growing steadily^1–3^. Glucocorticoids (GCs) such as Dexamethasone (Dex) are effective against many inflammatory conditions. However, two problems limit the use of GCs. On the one hand, chronic GC use causes serious side effects, such as hyperglycemia, growth arrest, glaucoma and skin thinning^4^. On the other hand, certain groups of patients do not respond to GC therapy^5,6^. Both issues are being addressed by searching for new molecules with enhanced GC therapeutic activities and fewer or less severe side effects.

GCs are lipophilic, so they penetrate the cell membrane and bind the GC receptor (GR). This receptor resides mainly in the cytoplasm. When the GR binds GCs, it translocates to the nucleus, where it performs its anti-inflammatory functions both as a monomeric protein and as a homodimer. The monomer is mainly known for transcriptional transrepression (TR), with the inflammatory transcription factors NF-κB and AP-1 as important targets, while the GR homodimer mediates transactivation (TA) to directly control DNA-dependent gene expression^7,8^. Although important anti-inflammatory genes are transcribed in response to the activities of dimeric GR (*e*.*g*., *DUSP1*, *IκBα*), there are metabolic side effects connected to this mechanism. Over the past decades, researchers have tried to develop Selective GR Agonists and Modulators (SEGRAM) hereby gradually moving away from steroidal scaffolds to nonsteroidal chemical entities^9^. More specifically, the development of Selective Monomer GR Agonists and Modulators (SEMOGRAM) aimed at favoring GR monomers over GR dimers is still regarded as a potential and viable strategy to separate the GR-mediated anti-inflammatory effects from the undesirable side effects. However, although several selective GR-triggering molecules have been developed and tested in clinical trials, no oral formulations have reached the market^9–11^.

In the transgenic mouse called GR^dim^ mouse, the GR has a single amino acid mutation (A > T) in the D-loop of the DNA binding domain (DBD) which contributes to homodimerization. Hence, in these mice, the dimerizing ability of the GR is compromised, leading to reduced GR-DNA binding^12^. A second dimerization interface, I634, has also been identified in the ligand binding domain (LBD)^13,14^. GR^dim^ mice are extremely sensitive to acute inflammatory conditions, such as lethal inflammatory shock induced by a single injection of lipopolysaccharides (LPS, endotoxin) or Tumor Necrosis Factor (TNF)^15–17^. Moreover, even the strong GR agonist Dex cannot protect these mice against LPS or TNF^18^. One obvious potential interpretation of these observations is that GR homodimerization is essential for protection, at least in acute inflammatory conditions. Along the same lines, we reason that GC resistance (GCR), as observed in the most acute and severe forms of inflammation (*e*.*g*., 100% GCR in systemic inflammatory response syndrome, SIRS, and sepsis), might be overcome by GCs that are more effective promoters of homodimerization^19^.

We set up a screening pipeline to identify Selective Dimerizing GR Agonists and Modulators (SEDIGRAM). Based on the literature, we selected several compounds, including the classical glucocorticoids Dex and Prednisolone^20^, the previously described TR-favoring nonsteroidal GR modulators (Fosdagrocorat^21^, Mapracorat^22^, LGD5552^23^), and the high affinity and full GR agonists Cortivazol^24,25^ and AZD2906^26,27^. By comparing the extent to which the different molecules consistently favored the formation of dimers and were biased towards TA rather than TR, we selected Cortivazol and AZD2906 as GR dimer-enhancing compounds that concomitantly lead to increased TA-dependent GR-mediated gene transcription. In support of our hypothesis predicting that Cortivazol and AZD2906 can provide enhanced therapeutic benefit in acute inflammation, both of them conferred stronger protection compared to Dex against the lethal effect of an aggressive systemic TNF-induced inflammatory insult *in vivo*. We further show that pharmacokinetic (PK) profiling may represent a necessary step in final selection of useful compounds for *in vivo* application.

## Results

### Increased GRE gene expression is representative of better GR dimerization

**Figure 1:**
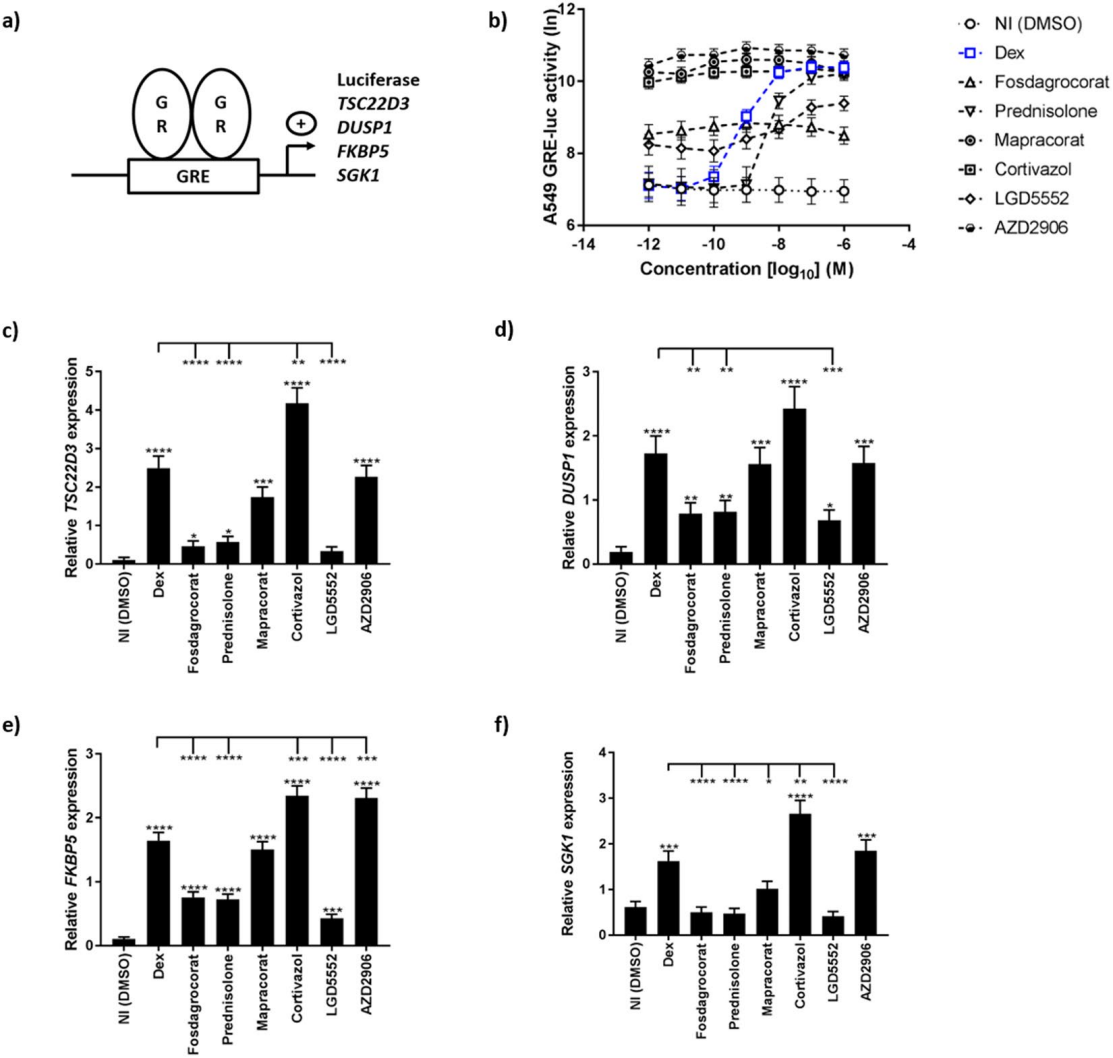
GRE-based reporter gene analysis and mRNA induction identify Cortivazol and AZD2906 as strong transactivating GR ligands. (**a**) *Schematic overview of GRE transcription principle*. (**b**) *GRE-luciferase assay*. A549 GRE-luc cells were stimulated with a dilution series of compounds for 5 h, after which they were lysed and luminescence was read out. (**c**–**f**) *Endogenous GRE gene-expression*. A549 GRE-luc cells were stimulated with 10^−8^ M of the compounds (5 h). mRNA expression of the genes (**c**) *TSD22D3*, (**d**) *DUSP1*, (**e**) *FKBP5* and (**f**) *SGK1* was measured by RT-qPCR. Data are shown as means ± SEM. A Hierarchical Generalized Linear Mixed Model (HGLMM) was fitted to the luciferase activities (measured at various concentrations of the compounds) and to the expression data. T statistics (unpaired, two-tailed) were used in (**b**) to assess the significance of fixed compound and concentration effects estimated as differences (on the transformed scale) to Dex at a particular concentration set as reference, in (**c**–**f**) to assess the significance of fixed compound effects estimated as differences (on the transformed scale) to the NI condition (significance levels above error bars) or Dex (significance indicated with−). Estimated mean values were obtained as predictions from the HGLMM, formed on the log scale (**b**) or on the scale of the response variable (**c**–**f**). To minimize graph complexity in (**b**) statistical differences are listed in Supplemental Table S5. P-values < 0.05, 0.01, 0.001 and 0.0001 are represented by *, **, *** or **** respectively. Non-significant differences are not indicated on the graphs. Experiments were repeated at least twice with at least two technical replicates included. NI: Non-Induced, Dex: Dexamethasone, GR: Glucocorticoid Receptor, GRE: Glucocorticoid Responsive Element.

Classical palindromic Glucocorticoid Responsive Elements (GRE) are DNA elements that bind GR homodimers. The canonical sequence of a GRE element is AGAACA(N)_3_TGTTCT^28–30^. After the agonist binds the GR LBD, formation of the dimerization interface is induced, and helix 12 is exposed. This leads to the binding of transcriptional coactivators, activation of RNA polymerase II and transcription of numerous genes^13,31,32^. For a given palindromic GRE, transcription stimulated by a particular GR ligand is thus likely to reflect the transcriptional activity of dimeric GR (Fig. 1a).

To test a SEDIGRAM-screening pipeline, six compounds were selected and compared to Dex, which we considered as a benchmark. Dex is a strong classical synthetic steroid 25 times more effective than hydrocortisone^33^. Compounds were selected based on frequent clinical use (Prednisolone is six times less effective than Dex^33^), based on previously described TA versus TR-dissociating activities (Fosdagrocorat^21,34^, Mapracorat^22^ and LGD5552^23,35^, representing negative selectors^9^), or based on their previously described high affinity and strong GR agonist profile (Cortivazol^24,25^ and AZD2906^26,27^).

All the compounds were first tested in an A549 GRE-luciferase (luc) reporter assay. The cells were stimulated for 5 h with a dilution series of the compounds and their respective DMSO solvent controls (NI, non-induced). Figure 1b shows that Dex induces maximal luciferase activity in the range of 10^−6^ M–10^−8^ M. In the same range, AZD2906 is more effective than Dex, while the others are equally effective (Mapracorat and Cortivazol) or less effective (Fosdagrocorat, Prednisolone, LGD5552). Also, all the compounds (except for Prednisolone) were remarkably more potent than Dex and were biologically active in minute concentrations. The dependency for GR in this biological system was confirmed using the GR antagonist RU486 and A549 GR-deficient GRE-luc cells (Supplemental Fig. S1).

After the initial screening with the GRE-luc reporter assay, we studied the induction of well-known GRE genes (*TSC22D3*, *DUSP1*, *FKBP5* and *SGK1*) in the same A549 GRE-luc cells using RT-qPCR analysis. Based on the GRE-luc curves, 10^−8^ M was chosen as an optimal and informative concentration at which Dex still has maximal activity. The cells were stimulated with the compounds for 5 h. Based on the RT-qPCR read-out, four compounds were identified as strong transcriptional inducers (Dex, Mapracorat, Cortivazol, AZD2906) and three as poor inducers (Fosdagrocorat, Prednisolone, LGD5552) (Fig. 1c–f). Cortivazol and AZD2906 were the only two compounds that induced more GRE-driven gene transcription than Dex, using different GRE-target genes as a read-out. Although the efficacies of AZD2906, Mapracorat and Cortivazol at 10^−8^ M were very similar in the reporter gene assay, subtle gene-specific differences were noted. These can be explained by the differences in GRE sequences between these genes. Indeed, according to Meijsing *et al*.^36^, GRE sequences slightly influence the conformation of DNA-bound GR, leading to changes in GR-dependent transcriptional output. We conclude that the A549 GRE reporter assay identified several compounds that are equally or more effective than Dex, which was confirmed by GRE gene read-out. To verify whether these compounds are genuine SEDIGRAM, they have to be further tested for their dimer-skewing potential.

### Study of GR dimerization by Fluorescence Resonance Energy Transfer (FRET)

**Figure 2:**
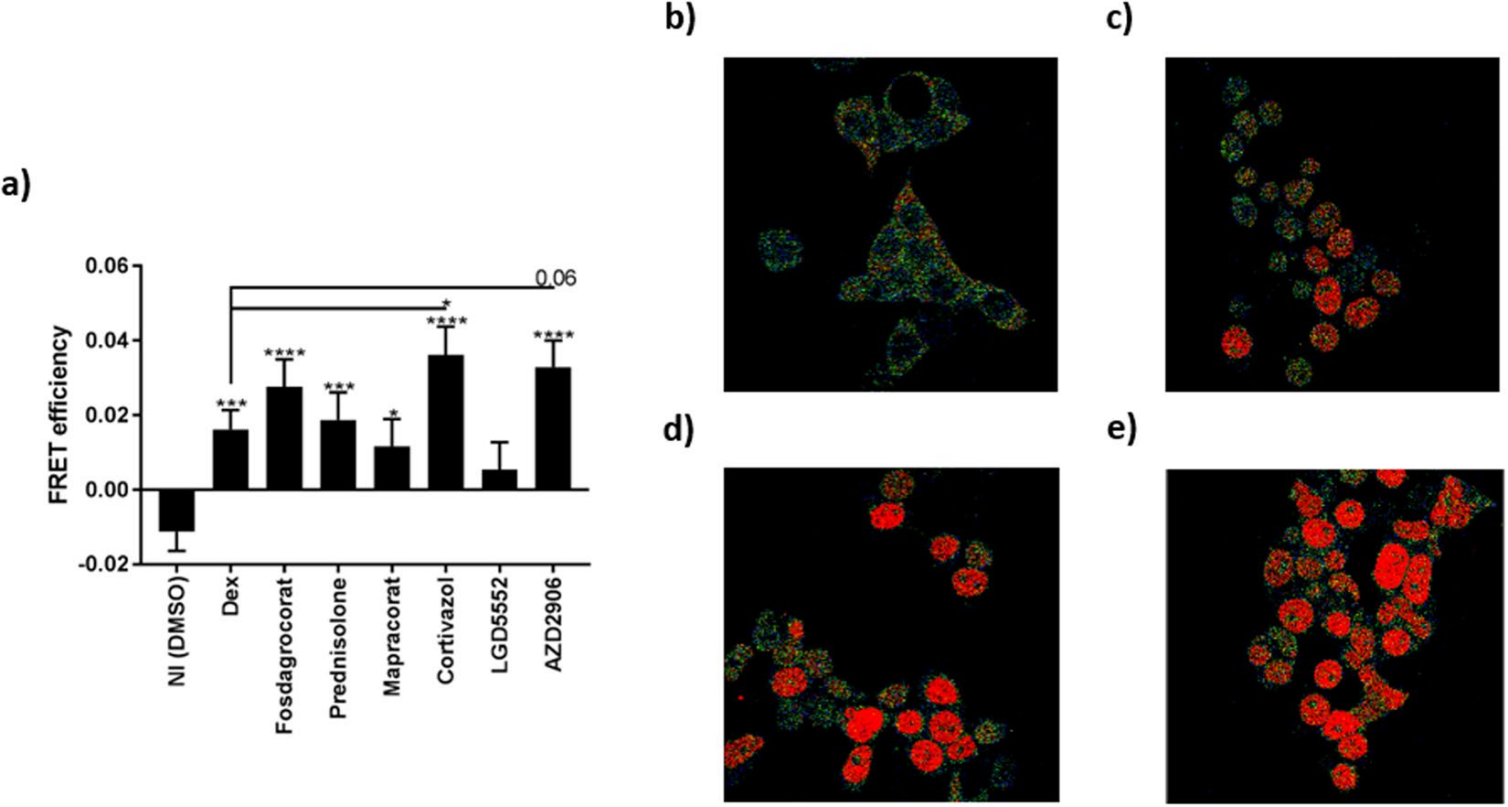
FRET identifies Cortivazol and AZD2906 as GR dimer-enhancing ligands. HEK293T cells were transfected with CFP-GR (donor) and YFP-GR (acceptor) and stimulated with 10^−8^ M of the compounds (1 h). (**a**) FRET efficiencies. (**b**) FRET image of NI cells. (**c**) FRET image of Dex stimulated cells. (**d**) FRET image of Cortivazol stimulated cells. (**e**) FRET image of AZD2906 stimulated cells. A Hierarchical Generalized Linear Mixed Model (HGLMM) was fitted to the FRET data. T statistics (unpaired, two-tailed) were used to assess the significance of fixed compound effects estimated as differences to the NI condition (significance levels above error bars) and Dex (significance levels indicated with−) set as reference. Estimated mean values were obtained as predictions from the HGLMM, formed on the scale of the response variable. P-values < 0.05, 0.01, 0.001 and 0.0001 are represented by *, **, *** or **** respectively. Non-significant differences are not indicated on the graph. Experiments were repeated at least twice with at least 15 technical replicates included. NI: Non-Induced, Dex: Dexamethasone.

The FRET technology was used to directly study the degree of GR dimerization after stimulation with the compounds. HEK293T cells co-transfected with CFP-GR (donor protein) and YFP-GR (acceptor protein) were stimulated with 10^−8^ M of the compounds for 1 h, after which FRET was studied. Figure 2 demonstrates that Cortivazol and AZD2906 induced more GR dimerization than Dex. This finding may explain how these two compounds increased GRE target gene transcription. Mapracorat, which generally induced similar levels of GRE genes as Dex, also induced GR-FRET to an extent resembling that of Dex. Consistently, under the same conditions, Fosdagrocorat, Prednisolone and LGD5552, all of which scored lower in TA efficacy than Dex (Fig. 1), did not show a lower degree of dimerization as compared to Dex (Fig. 2), suggesting that other factors contribute to their transactivating capacities. For example, Fosdagrocorat induced a substantial degree of GR dimerization (Fig. 2) but did not score high on the different TA read-outs (Fig. 1). From these data, it also seems that LGD5552 may be the only compound with genuine GR-dimer compromising activities. Together, the data in Figs 1 and 2 show that Cortivazol and AZD2906 are promising SEDIGRAM compounds that induce increased GR dimerization and concomitantly possess enhanced TA capacity.

### Effect of compounds on TNF-induced NF-κB luciferase activity

**Figure 3:**
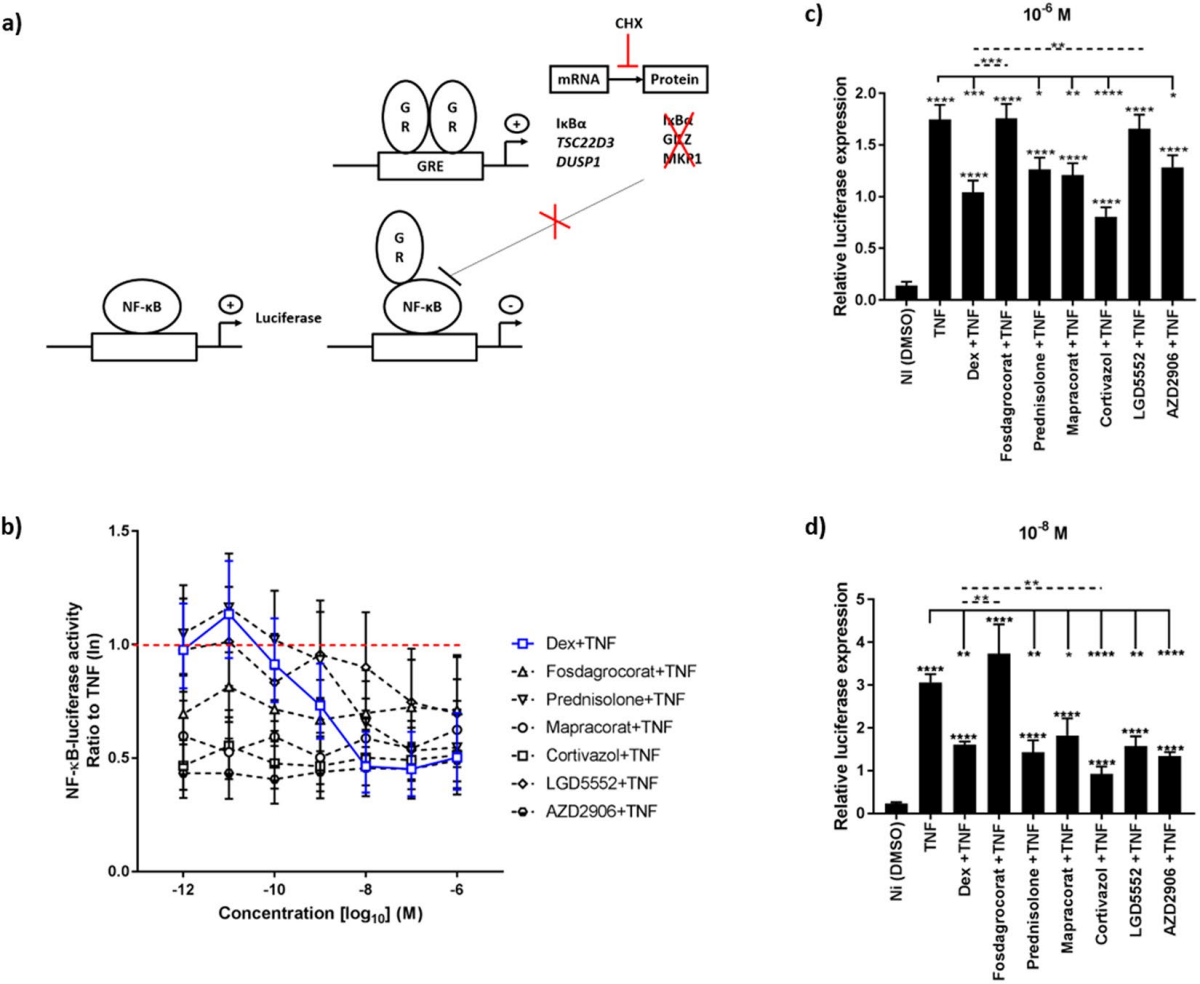
GR dimer-favoring compounds still support transrepression of NF-κB in the absence of *de novo* protein synthesis. (**a**) Schematic overview of the NF-κB-luciferase and cycloheximide (CHX) principle. (**b**) NF-κB-luciferase activity. A549 NF-κB-luc cells were pretreated with a concentration range of the compounds (1 h), followed by challenge with 1000 U/ml hTNF for 5 h, after which they were lysed and luminescence was read out. The graph represents the ratio to TNF (red dotted−). (**c**,**d**) NF-κB-luciferase gene expression. A549 NF-κB-luc cells were pretreated with 10 µg/ml CHX (30 min), followed by stimulation with 10^−6^ M (**c**) or 10^−8^ M (**d**) of the compounds. After 1 h, the cells were challenged with 1000 U/ml hTNF for 2 h. Data are shown as mean ± SEM. A Hierarchical Generalized Linear Mixed Model (HGLMM) was fitted to the luciferase activities (measured at various concentrations of the compounds) and to the expression data. T statistics (unpaired, two-tailed) were used in (**b**) to assess the significance of fixed compound and concentration effects estimated as differences (on the transformed scale) to Dex at a particular concentration set as reference, in (**c**,**d**) to assess the significance of fixed compound effects estimated as differences (on the transformed scale) to NI (significances above error bars), TNF (indicated with−) or Dex (indicated with dotted−) set as reference. Estimated mean values were obtained as predictions from the HGLMM, formed on the log scale (**b**) or on the scale of the response variable (**c**,**d**). To minimize graph complexity in (**b**) statistical differences are listed in Supplemental Table S6. P-values < 0.05, 0.01, 0.001 and 0.0001 are represented by *, **, *** or **** respectively. Non-significant differences are not indicated on the graph. Experiments were repeated at least twice with at least two technical replicates included. NI: Non-Induced, TNF: Tumor Necrosis Factor, Dex: Dexamethasone, GR: Glucocorticoid Receptor, GRE: Glucocorticoid Responsive Element.

An essential need within the SEDIGRAM screening pipeline is to identify molecules not only with an increased GR dimer profile in line with increased TA abilities, but preferably, also with a target gene-selective profile shifting the balance towards the GR dimer and less towards GR monomer regulated genes. Studies using the GR^dim^ mutant mice, with compromised GR dimerization and TA, showed that AP-1 driven TR mechanisms are preserved^12^. Along the same lines, a plant-derived compound favoring GR-mediated TR of NF-κB-driven genes^37^ was shown by different technologies to support GR monomer formation within a cellular environment^14,38,39^. For these reasons, based on the above findings, we evaluated to what extent the selected compounds support TR, as a mechanism linked to GR monomer activity (see scheme Fig. 3a). Stably transfected A549 NF-κB-luc cells were stimulated with human TNF (hTNF) for 5 h with or without pretreatment of the compounds for 1 h. At the concentrations at which Dex is maximally repressing the TNF-induced NF-κB-luc activity (10^−6^ M–10^−8^ M) none of the compounds is able to surpass Dex in its TR efficacy (Fig. 3b). However, most compounds (Fosdagrocorat, Mapracorat, Cortivazol and AZD2906) do show a markedly higher potency to transrepress as compared to Dex at very low doses, i.e. 10^−9^ M and lower.

Although this luciferase assay is a first good assessment of potential GR monomer activity, it must be taken into account that secondary effects resulting from endogenous GRE gene products (induced by the GR dimer) may also contribute to inhibition of NF-κB activity: dimeric GR may induce e.g. the inhibitor of NF-κB (*IκBα*)^40,41^, *TSC22D3*^42^ and *DUSP1*^43^, all of which can block NF-κB. To rule out such effects, the cells were pretreated for 30 min with cycloheximide (CHX) to block mRNA translation, and thus the contribution of anti-inflammatory protein production, following the induction of GRE genes (principle see scheme Fig. 3a). Inhibition of protein synthesis by CHX was verified as shown in Supplemental Fig. S2. Because transcription is not inhibited by CHX, luciferase mRNA levels could be measured by RT-qPCR. We determined compound effects at 10^−6^ M and 10^−8^ M (Fig. 3c,d). The data show that, in the presence of CHX, Fosdagrocorat and LGD5552 appear as the least potent NF-κB transrepressing molecules. Cortivazol suppressed NF-κB-luc transcription more efficiently than Dex (Fig. 3c,d and Supplemental Fig. S3). This result may either imply that enhanced GR dimer formation can also be compatible with enhanced TR or else, that the NF-κB interaction interface may still dictate disassembly of GR dimer formation at the chromatin. Also, as a strong GR dimerizing compound, AZD2906 proved not to be better in repressing NF-κB-luc transcription than Dex (Figs 3c,d and S3). Together, these data suggest that AZD2906 might fit the profile of a SEDIGRAM slightly better than Cortivazol by supporting increased GR dimer formation and TA abilities as compared to Dex, yet performing not better than Dex in the monomer-linked TR assay.

### Assay to address whether SEDIGRAM candidates protect better against TNF-induced acute inflammation: induction of interleukin 6 (IL6)

**Figure 4:**
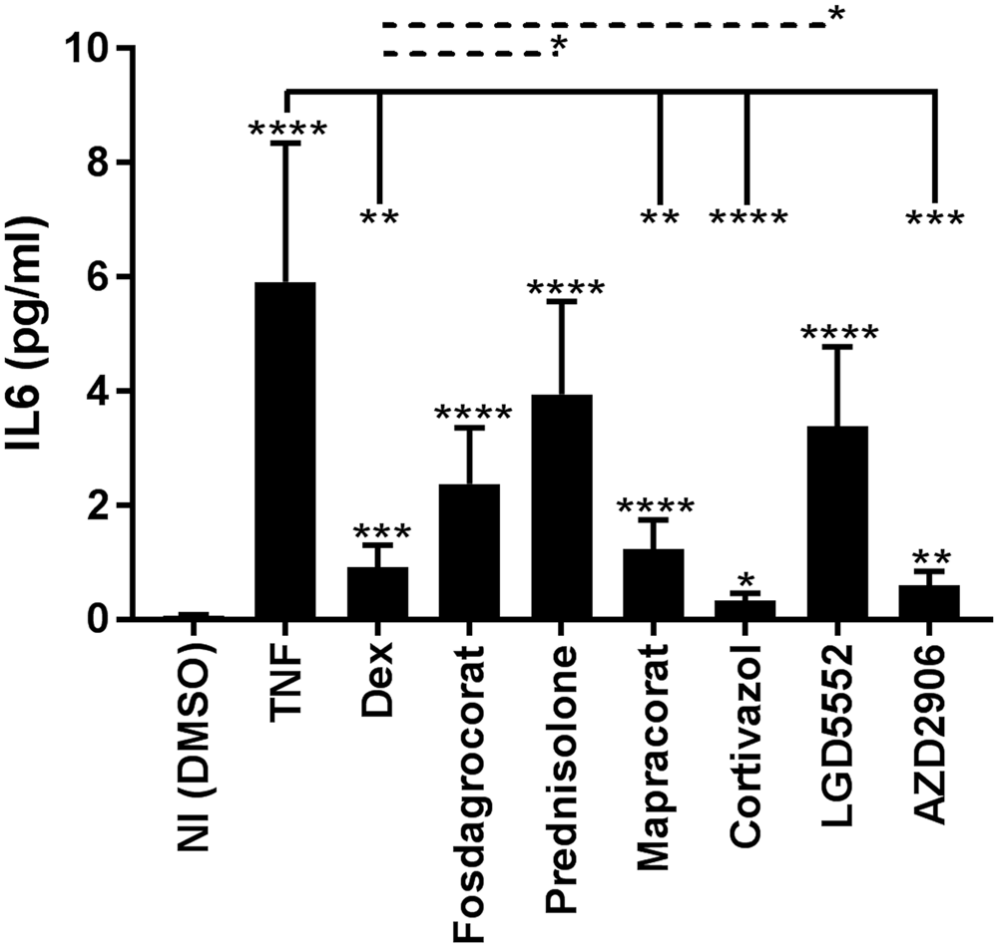
Dimerization correlates with effective anti-inflammatory activity *in vitro*. A549 GRE-luc cells were stimulated with the compounds (10^−8^ M) for 1 h, followed by challenge with 1000 U/ml hTNF. After 24 h, hIL6 levels were measured in the supernatant. Data are shown as mean ± SEM. A Hierarchical Generalized Linear Mixed Model (HGLMM) was fitted to the expression data. T statistics (unpaired, two-tailed) were used to assess the significance of fixed compound effects estimated as differences (on the transformed scale) to NI (significances above error bars), TNF (indicated with−) and Dex (indicated with dotted−) set as reference. Estimated mean values were obtained as predictions from the HGLMM, formed on the scale of the response variable. P-values < 0.05, 0.01, 0.001 and 0.0001 are represented by *, **, *** or **** respectively. Non-significant differences are not indicated on the graph. The experiment was repeated twice with at least three technical replicates included in the individual experiments. NI: Non-Induced, TNF: Tumor Necrosis Factor, Dex: Dexamethasone.

To investigate whether a link exists between GR dimerizing potential/TA output of the compounds and protection against TNF-induced acute inflammation, we first studied TNF-induced IL6 induction in an *in vitro* assay. We pretreated A549 GRE-luc cells for 1 h with 10^−8^ M of the compounds, stimulated the cells with hTNF for 24 h and studied the production of the inflammatory marker IL6. In Fig. 4, we display that Dex and Mapracorat which had equally efficient GR-dimerizing and GR-dimer dependent TA properties, have equal effects in this ‘*in vitro* inflammation’ test. Also, Cortivazol and AZD2906, the best candidate SEDIGRAM so far, most significantly repressed IL6 production, while other compounds inducing less GR TA (Fosdagrocorat, Prednisolone) were less efficient. Interestingly, the only monomer-supporting LGD5552 also showed a less efficient suppression of IL6. These data support the hypothesis that increasing the formation of GR dimers may be a valuable strategy to increase the efficiency of GR agonists against acute TNF-induced inflammation.

### The GR-dimer potentiating molecules Cortivazol and AZD2906 convey enhanced protection against acute TNF-induced SIRS *in vivo*

**Figure 5:**
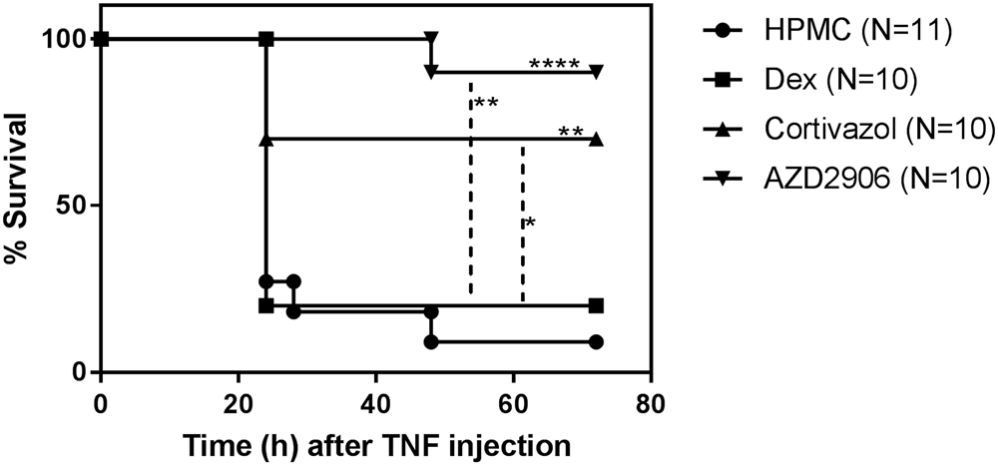
Cortivazol and AZD2906 are more protective than Dex against acute TNF-induced SIRS *in vivo*. Female C57BL/6J mice aged 8 weeks were gavaged with 1 mg/kg Dex or an equimolar dose of Cortivazol or AZD2906 dissolved in 100 µl HPMC. One hour after gavage, the mice were injected intraperitoneally with 50 µg mTNF per 20 g bodyweight. Survival was monitored. None of the mice died after 72 h after the TNF challenge. Survival curves were compared with a Mantel-Cox test. Significant differences compared to HPMC (above survival curves) and Dex (indicated with dotted−) are given. P-values < 0.05, 0.01, 0.001 and 0.0001 are represented by *, **, *** or **** respectively. Non-significant differences are not indicated on the graph. Results of two pooled experiments are shown. HPMC: Hydroxypropylmethylcellulose, Dex: Dexamethasone, TNF: Tumor Necrosis Factor.

A single administration of Dex protects against acute, lethal inflammation induced by a lethal dose of TNF^44^. GR^dim^ mice (expressing a GR protein with reduced dimerization) are significantly more sensitive to TNF induced SIRS^16^ and these mice cannot be protected against TNF by Dex^18^. We therefore reasoned that compounds with an enhanced GR dimer forming potential might be of interest in acute (TNF-induced or –mediated) inflammation. We tested this hypothesis by administrating 1 mg/kg Dex or an equimolar dose of Cortivazol or AZD2906 via oral gavage 1 h before a high and lethal intraperitoneal TNF injection. Under these conditions, the dose of Dex is unable to confer protection. Figure 5 clearly shows that mice treated with Cortivazol or AZD2906 experienced a significantly enhanced protection against TNF as compared to the Dex- or vehicle treated mice.

GC therapy is associated with several side effects^4,19^. Although side effects are most relevant during long-term treatment, their assessment is necessary even within acute applications. To have an idea of side effects, we treated mice with Dex, Cortivazol or AZD2906 by oral gavage, once a day during 5 consecutive days during which bodyweight was monitored. 6 h after the last gavage, mice were euthanized and liver, kidney, thymus and spleen were isolated and weighed. Supplemental Fig. S4 shows that all agonists decreased the total bodyweight as well as the weight of the spleen. Cortivazol and AZD2906 did not significantly exceed the effect of Dex. All ligands increased liver weight, and we found a significantly more severe effect with AZD2906 compared to Dex.

### Increased protection in acute inflammation by Cortivazol and AZD2906 coincides with a shift in the PK profile as compared to Dex

**Figure 6:**
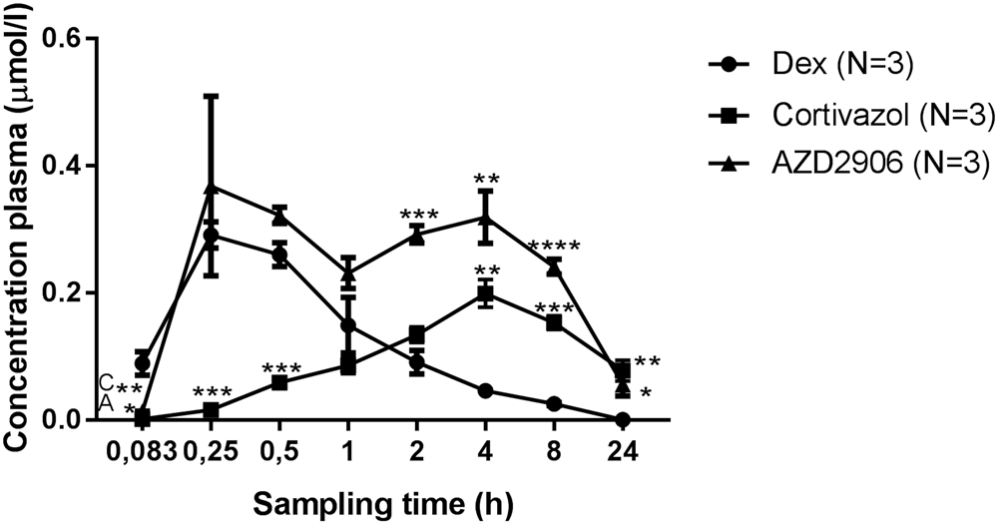
Cortivazol and AZD2906 are retained longer in the mouse. CD1 female mice were gavaged with 100 µl of 1 mg/kg Dex, Cortivazol or AZD2906 dissolved in HPMC. Blood was collected by retro-orbital bleeding and the concentrations of the compounds in plasma were determined by mass spectrometry analysis. Data are shown as mean ± SEM. Significant differences of Cortivazol and AZD2906 compared to Dex are represented in the graph and calculated with and unpaired, two-tailed T test at the indicated time points. P-values < 0.05, 0.01, 0.001 and 0.0001 are represented with *, **, *** or **** respectively. Non-statistical differences are not indicated on the graph. Dex: Dexamethasone, C: Cortivazol, A: AZD2906, HPMC: Hydroxypropylmethylcellulose.

A PK profiling was performed in the plasma of mice given an oral gavage of 1 mg/kg Dex, Cortivazol or AZD2906. Plasma compound concentrations were measured by mass spectrometry (MS) over a 24 h timeframe. As seen in Fig. 6, Dex peaks after 15 min-30 min in the plasma, while Cortivazol obtains plasma peak levels after 4 h. The PK profile of AZD2906 is peculiar since this molecule displays a biphasic pattern, with a first peak after 15 min-30 min and a second one after 4 h. The data show that the two compounds found in the *in vitro* SEDIGRAM screen lead to better anti-inflammatory effects *in vivo*, but also to delayed PK clearance. These data would suggest that the GR-dimer favoring molecules able to display improved anti-inflammatory effects *in vivo*, might be doing so because of a delayed PK clearance. We confirmed that increased dimerization has no effect on the PK of the compounds by making use of GR^dim^ mice (data not shown).

## Discussion

GCs are among the most effective drugs widely used to combat inflammation. However, side effects such as hyperglycemia, growth arrest, glaucoma and skin thinning limit their use, particularly after long-term treatment^4^. Moreover, some patients are resistant to GC therapy^5,6^. For decades, researchers have been looking for new GC molecules and numerous screening and scaffolding studies have been trying to look for or design GR ligands that are highly GR-specific, potent and selective^26,27,45–47^. Compound design is guided by new insights into GR biology and over recent years we have learned some important lessons on the effects of ligand-LBD interactions on GR activity. GCs and other GR ligands bind the LBD pocket and, depending on their orientation and interactions, induce a GR conformational change. This change determines which transcriptional coregulators will be recruited, DNA binding, and the nature and intensity of the resulting GR transcriptional response^13,31,32,48,49^. Interaction of the ligand with helices 3 and 5 and the positioning of helix 12, which together form the LBD pocket, are crucial for receptor activation^50–52^. Intense research efforts went into the discovery and development of SEMOGRAM with the aim of separating the beneficial anti-inflammatory TR effects from the TA-induced side effects of GCs^9–11,53,54^. Unfortunately, for several reasons^19^, none of the proposed SEMOGRAM is available on the market for systemic use. First, dissociating GR TA from TR to separate the GC side effects from the anti-inflammatory effects is an overly simplified model because particular side effects are still found in GR^dim^ mice and thus are considered as monomer-dependent^55,56^. Second, most of the research focusing on decreased GR TA may have been complicated by a simultaneous loss of TR resulting in generally less performant GCs (as is the case of Fosdagrocorat)^21,34^. Third, the contribution of the induction of GR dimers and thus TA of GRE-containing genes coding for anti-inflammatory proteins, such as *TSC22D3* and *DUSP1*, has been widely underestimated in the anti-inflammatory success of GCs^57–61^. Evidence for this can be found in studies on GR^dim^ mice. These mice carry an A > T point mutation in the D-loop of the DNA-binding domain of GR and are characterized by weaker GR dimerization, weaker DNA binding and diminished GRE gene transcription, but GR TR remains intact^12,62^. In acute inflammatory settings such as lethal inflammatory shock induced by TNF^16^ or LPS^15,17^ GR^dim^ mice are extremely sensitive and GCs fail to confer protection^18^. The interpretation that protection against acute inflammation by GCs is largely or exclusively driven by GR dimers^19^ was confirmed by studies using CpdA, a plant-derived non-steroidal SEMOGRAM^38,63^. CpdA was unable to protect against the inflammatory response in the acute TNF *in vivo* model. Rather, CpdA sensitized mice in this model due to its ability to selectively induce GR monomers and to favor TR of NF-κB. As such, CpdA sensitized for TNF-induced inflammation in a JNK2-mediated way, which is normally blocked by the GR dimer-induced protein MKP1^63^. Other studies indeed have shown that CpdA inhibits GR homodimerization^39^.

None of the screening or scaffolding studies so far have focused on ligands increasing GR dimer formation. In this study, we aimed to screen for SEDIGRAM exhibiting enhanced TA abilities as well as a more prominent GR dimerization profile. We established a confidence-building screening pipeline to compare GR dimer with GR monomer activity. We complemented GRE-luc-based TA assays with a four-panel GRE-target gene read-out and FRET, the latter enabling direct assessment of dimerization. Moreover, we used adjusted TR assays that exclude a possible contribution of secondary GR TA and thus GRE mediated TR (*e*.*g*., IκBα-mediated repression of NF-κB). Current SEMOGRAM research might have underestimated the contribution of the GR dimer to the anti-inflammatory effects of some of these intensively studied agonists. For example, the anti-inflammatory protection provided by Mapracorat was initially ascribed to its TR activity. Mapracorat, first described by Schäcke *et al*.^22^, is a nonsteroidal dissociative GR agonist with an improved side effect profile and with therapeutic opportunities for inflammatory eye and skin diseases. Although Mapracorat showed less activity in the TA studies of Schäcke *et al*.^22^, later research showed that its anti-inflammatory potential is probably due to inhibition of Mitogen Activated Protein Kinases via the induction of *DUSP1* (or MKP1), and hence on TA^58,59^. Based on our results, Fosdagrocorat may be a similar case.

**Figure 7:**
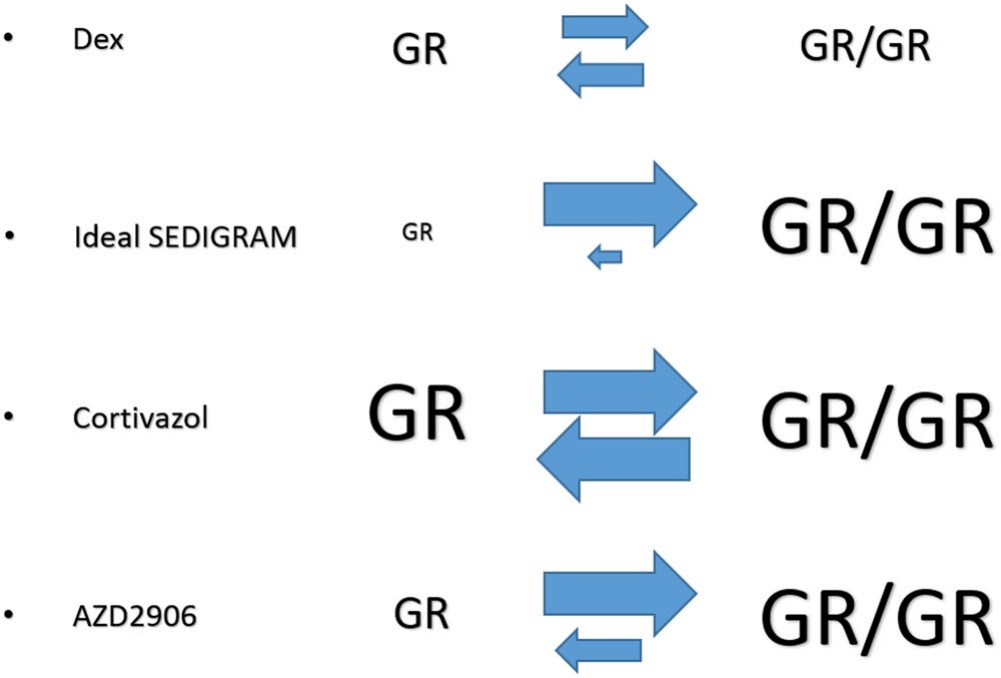
Concluding scheme of the study. Dex, a benchmark glucocorticoid, is able to induce both GR monomers (depicted as GR) and dimers (depicted as GR/GR). An ideal SEDIGRAM is defined as a compound that favors dimerization at the cost of monomers. Cortivazol increases both GR monomer and GR dimer activity and is therefore a generally stronger GR agonist. AZD2906 specifically increased the GR dimer activity compared to Dex while leaving GR monomer activity untouched, and therefore leans closer to the SEDIGRAM concept.

From the small group of compounds we tested, we were able to select Cortivazol and AZD2906 as having enhanced GR-dimerizing and GRE-TA agonist profiles compared to the benchmark GC Dex (Table 1 and Fig. 7 provide a general overview of compound performances compared to Dex). Cortivazol is a highly potent steroidal GC. It differs from standard GCs in that it can interact with two sites in the GR protein, whereas classical GCs have only one binding site. Yoshikawa *et al*.^64^ reported that Cortivazol induces conformational changes in the LBD, reorienting several amino acids and making additional contacts with helices 3 and 5, which stabilizes the LBD configuration. Cortivazol was further shown to be a selective modulator of GR in the sense that its transcriptome only partly overlaps with that of Dex. The latter study^65^ linked specific Cortivazol-regulated genes to its ability to induce apoptosis in leukemia cells that are resistant to Dex. Although apoptosis is different from anti-inflammatory regulation, together with our results, these data suggest that Cortivazol might be useful in GCR inflammatory diseases such as SIRS and sepsis. AZD2906 is a nonsteroidal, full agonist of the GR developed by AstraZeneca. It exhibits full efficacy in the TA assay and provides good protection in a rat model of joint inflammation. Like Cortivazol, AZD2906 is optimally oriented in the LBD for receptor activation^26,27^. Cortivazol and AZD2906 might be useful starting molecules because their LBD interactions might influence GR dimerization. AZD2906 originated from a drug scaffolding series aimed at developing more potent compounds. The chemical groups thus increase not only potency, but also GR dimerization. In general, new knowledge about the effect of the ligand backbone orientation and ligand side group interactions in the LBD pocket, together with new insights in GR dimer biology, could lay the ground for developing new scaffolding strategies for future SEDIGRAM development.

**Table 1:** General overview of compound induced Glucocorticoid Receptor activity relative to Dexamethasone. Compound effects per (*in vitro*) experiment relative to Dexamethasone (100%) are presented in the table. E_max_: maximal efficacy, GRE: Glucocorticoid Responsive Element, FRET: Fluorescence Resonance Energy Transfer, ELISA: Enzyme-Linked Immunosorbent Assay.

		Fosdagrocorat	Prednisolone	Mapracorat	Cortivazol	LGD5552	AZD2906
GRE-luciferase (E_max_)		21.04	82.82	123.02	96.01	36.55	171.03
Endogenous GRE	*SGK1*	30.64	29.29	62.37	163.69	25.46	113.75
	*TSC22D3*	18.70	22.93	69.80	167.41	13.37	90.88
	*DUSP1*	45.56	47.35	90.48	140.74	39.64	91.34
	*FKBP5*	46.08	44.09	91.64	142.71	26.07	140.55
FRET		169.90	115.35	72.13	222.82	33.32	202.22
NF-κB-luciferase (E_max_)		58.21	91.10	75.49	97.94	61.16	102.59
NF-κB-luciferase 10^−6^ M		0.00	68.91	76.27	133.50	12.77	65.76
NF-κB-luciferase 10^−8^ M		0.00	117.34	92.74	143.92	108.45	117.03
IL6 ELISA		70.71	39.43	93.67	111.85	50.63	106.49

We further showed that it is useful to complement the screening assays with PK testing. Here, the pre-selected compounds were not only more protective than Dex in the *in vivo* TNF-induced lethal SIRS model, both compounds were cleared more slowly, which could have contributed to the protection they provided *in vivo*. Although the relative contributions of increased GR dimerization and decreased plasma clearance to the protection against SIRS is not clear, our screening pipeline identified two compounds that are promising therapeutic candidates meriting further attention in the context of acute inflammation.

GCR is a major problem in the management of inflammatory diseases. Several cytokines such as TNF have been shown to induce GCR *in vitro* and *in vivo*^44,66,67^. Several strategies to overcome GCR can be followed, such as reverting the resistance or making use of GR ligands that are unaffected. However, Rider *et al*.^66^ showed that the magnitude of the effect of TNF on the TA of partial or full agonists of the GR was the same. In other words, although GR activity induced by more effective ligands was affected by TNF to the same extent as less effective ligands, more efficacious ligands can still induce a certain degree of GR TA due to their superiority in GRE gene induction efficiency. There is a need to investigate whether strong SEDIGRAM can overcome or resist GCR, which occurs particularly during exacerbation of inflammation and in severe cases of inflammation^5^. Studies on GR^dim^ mice suggest that GR dimer activity is absolutely necessary in the protection against severe acute inflammation. The apoptotic activity of Cortivazol in Dex-resistant leukemia cells is promising^65,68^.

Since certain important GR side effects (*e*.*g*., thymocyte apoptosis) are directly linked to GR TA^8^, one might question the therapeutic applicability of SEDIGRAM compounds. Here we assessed GC-induced acute side effects and found that Cortivazol and AZD2906, like Dex, also changed several of the parameters that we measured. A previous report also mentioned AZD2906 was not progressed further despite its good protection in a joint inflammation model, because it was found to have limited advantage compared to Prednisolone in regard to its side effect profile^27^. Although care must be taken, it is unlikely that these side effects outweigh the therapeutic benefit of using SEDIGRAM in life-threatening cases of acute inflammation, such as shock. Besides, as SEDIGRAM are meant for treatment of severe acute inflammation, which is most often treated in intensive care units, acute side effects such as hyperglycemia are supposed to be limited and controllable.

In conclusion, we have developed a pipeline to screen for robust SEDIGRAM and characterized two compounds, Cortivazol and AZD2906. Although both these ligands induce more GR dimerization and TA than Dex, the ideal SEDIGRAM were not identified in this study since GR monomer activity was still highly activated. Our data suggest that Cortivazol even increased GR monomer activation and that it is therefore rather an extremely active GR agonist. AZD2906 on the other hand is closer to the ideal SEDIGRAM profile, because our data suggest that only its TA capacity is increased compared to Dex, while its degree of activation of GR monomer activity is similar as Dex (principles shown in Fig. 7). Both Cortivazol and AZD2906 provided more therapeutic benefit than the benchmark Dex in a mouse model of TNF-induced severe acute inflammation. Because both compounds also have a slower plasma clearance, the extent to which the superiority of these compounds is due to their increased GR dimerization potential remains to be investigated. We believe that our work emphasizes that the search for and development of SEDIGRAM with maximal dimer-promoting activity might hold the key to the development of therapeutically useful drugs for unmet medical needs such as SIRS and sepsis.

## Methods

### Cell culture

A549 and HEK293T cells were maintained and grown in “supplemented DMEM” medium, i.e. Dulbecco’s modified Eagle’s medium (DMEM, house-made) containing 10% fetal calf serum, 1 mM sodium pyruvate, 0.1 mM non-essential amino acids and 2 mM L-glutamate, at 37 °C and 5% CO_2_.

### Plasmids

GRE-luc (p(GRE)_2_50hu.IL6P-luc+) and NF-κB-luc plasmids (p(IL6κB)_3_50hu.IL6P-luc+), and the stable integration in A549 cells, were previously described^69–71^. CFP-GR (pECFP-hGR) and YFP-GR (pEYFP-GR) plasmids were kindly provided by Dr. Ann Louw (Stellenbosch, Republic South Africa).

### Reagents

Dex, Prednisolone and RU486 were purchased from Sigma-Aldrich. Cortivazol and LGD5552 were purchased at Syncom. Fosdagrocorat, Mapracorat and AZD2906 were synthesized at Bioduro, GVK and Chiroblock respectively. All compounds were dissolved in dimethylsulfoxide (DMSO). Recombinant human and mouse TNF was expressed in and purified from *E*. *Coli* in our department.

### Luciferase reporter assays

30.000 A549 GRE-luc or A549 NF-κB-luc cells were seeded per well in supplemented DMEM in a 96 well culture plate. 18 h later medium was replaced with Optimem (Gibco, Invitrogen). A549 GRE-luc cells were stimulated for 5 h with a serial dilution of compounds (10^−6^ M–10^−12^ M) or the respective amount of DMSO solvent control in Optimem. A549 NF-κB-luc cells were pretreated for 1 h with a serial dilution of compounds (10^−6^ M–10^−12^ M) or solvent control after which they were challenged by adding 1000 U/ml hTNF for 5 h. Cells were harvested, lysed (25 mM Tris-phosphate pH 7.8, 2 mM dithiothreitol, 2 mM 1.2-diaminocyclohexane-N,N,N′,N′-tetraacetic acid, 10% glycerol, 1% Triton X-100) and luciferase activity was evaluated by measuring the D-luciferin (L-1349, Duchefa) conversion (Glomax, Promega).

### RNA isolations and RT-qPCR

500.000 A549 GRE-luc or A549 NF-κB-luc cells were seeded per well in supplemented DMEM in 6 well culture plates. 18 h later medium was replaced with Optimem. A549 GRE-luc cells were stimulated for 5 h with 10^−8^ M of the compounds or the solvent control for GRE gene expression analysis. A549 NF-κB-luc cells were pretreated for 30 min with 10 μg/ml CHX (C7698, Sigma-Aldrich), followed by a 1 h stimulation with 10^−6^ M or 10^−8^ M of the compounds or solvent control and subsequently stimulated with 1000 U/ml hTNF for 2 h. Total RNA was isolated using TRIzol (Gibco, Life Technologies) and the Invitrap Spin Universal RNA Mini Kit (Invitek, Isogen Life Science) according to the manufacturer’s instructions. RNA concentrations were measured using Nanodrop 1000 (Thermo Scientific). 1000 ng RNA was used for cDNA synthesis (iScript Advanced cDNA Synthesis Kit, Bio-Rad). qPCR (LightCycler 480, Roche) was performed with the SensiFast SYBR No-ROX kit (Bioline). The following primers were used: *TSC22D3* (forward 5′-GGAGATCCTGAAGGAGCAGA-3′, reverse 5′-TTCAGGGCTCAGACAGGACT-3′), *DUSP1* (forward 5′-ACCACCACCGTGTTCAACTTC-3′, reverse 5′-TGGGAGAGGTCGTAATGGGG-3′), *FKBP5* (forward 5′-GCCACATCTCTGCAGTCAAA-3′, reverse 5′-TCCCTCGAATGCAACTCTCT-3′), *SGK1* (forward 5′-CCTCCACCAAGTCCTTCTCA-3′, reverse 5′-CCCTTTCCGATCACTTTCAA-3′) and *luciferase* (forward 5′-ATACAAAGGATATCAGGTGG-3′, reverse 5′-TTGCGTCGAGTTTTCCGG-3′). Expression levels were normalized to the expression levels of the housekeeping genes *36B4* (forward 5′-CATGCTCAACATCTCCCCCTTCTCC-3′, reverse 5′-GGGAAGGTGTAATCCGTCTCCACAG-3′) and Cyclophilin A (forward 5′-TCCTGGCATCTTGTCCATG-3′, reverse 5′-CCATCCAACCACTCAGTCTTG-3′) which were determined with the geNorm Housekeeping Gene Selection Software (Biogazelle, Belgium).

### FRET

20.000 HEK293T cells were seeded per well in supplemented DMEM in μ-Slide 8 Well ibiTreat plates (Ibidi). 350 ng CFP-GR and 300 ng YFP-GR were transfected using CaPO_4_ transfection. 6 h later medium was changed to Optimem. After 24 h, cells were stimulated for 1 h with 10^−8^ M of the compounds or the solvent control. FRET was determined using an LSM780 multiphoton confocal microscope (Zeiss) equipped with a temperature (37 °C) and CO_2_ (5%) controlled chamber. A Plan-Apochromat 63x/1.40 Oil DIC M27 objective lens was used to acquire 16-bit images at a resolution of 1024 by 1024 pixels (pixel size = 132 nm). CFP-GR was excited with a TiSa multiphoton laser tuned to 800 nm at 3,5%. CFP-GR (donor) emission was detected by PMT1 between 438 and 515 nm, gain set at 850. FRET emission was detected by a QUASAR detection unit in the range from 525 to 630 nm, gain set at 740. YFP-GR (acceptor) was excited with a multi-Argon laser using the 514 nm laser line, at a laser power of 0,3%. YFP-GR emission was detected similarly by the QUASAR unit using identical settings. The signals measured in the FRET channel were corrected for cross-talk and cross-excitation from the cyan and yellow channels using the following equation for apparent FRET efficiency described by van Rheenen *et al*.^72^:

$$\begin{array}{ccc}{\rm{E}}{\rm{A}}{\rm{i}} & = & ({\rm{S}}{\rm{i}}{\rm{g}}{\rm{n}}{\rm{a}}{\rm{l}}\,{\rm{I}}{\rm{n}}{\rm{d}}{\rm{i}}{\rm{r}}{\rm{e}}{\rm{c}}{\rm{t}}\,{\rm{A}}{\rm{c}}{\rm{c}}{\rm{e}}{\rm{p}}{\rm{t}}{\rm{o}}{\rm{r}}-({\rm{S}}{\rm{i}}{\rm{g}}{\rm{n}}{\rm{a}}{\rm{l}}\,{\rm{D}}{\rm{o}}{\rm{n}}{\rm{o}}{\rm{r}}\,\ast \,\beta )\\  &  & -({\rm{S}}{\rm{i}}{\rm{g}}{\rm{n}}{\rm{a}}{\rm{l}}\,{\rm{D}}{\rm{i}}{\rm{r}}{\rm{e}}{\rm{c}}{\rm{t}}\,{\rm{A}}{\rm{c}}{\rm{c}}{\rm{e}}{\rm{p}}{\rm{t}}{\rm{o}}{\rm{r}}\ast (\gamma -\alpha \ast \beta )))\\  &  & /({\rm{S}}{\rm{i}}{\rm{g}}{\rm{n}}{\rm{a}}{\rm{l}}\,{\rm{D}}{\rm{i}}{\rm{r}}{\rm{e}}{\rm{c}}{\rm{t}}\,{\rm{A}}{\rm{c}}{\rm{c}}{\rm{e}}{\rm{p}}{\rm{t}}{\rm{o}}{\rm{r}}\ast (1-\beta \ast \delta ))\end{array}$$

with α, β, γ and δ being correction factors for cross-excitation and cross-talk calculated from specimens expressing one construct only.

### hIL6 ELISA

hIL6 levels were measured in the supernatant of A549 GRE-luc cells using the human IL6 ELISA ready-set-go kit (Invitrogen, eBioscience) according to the manufacturer’s instructions. Cells were seeded as described for qPCR purposes and were pretreated for 1 h with 10^−8^ M of the compounds or the solvent control and subsequently challenged with 1000 U/ml hTNF. Supernatant was collected 24 h later.

### Mice

For survival testing 8 week old female C57BL/6 J mice were purchased from Janvier (Le Genest-St.Isle, France). Mice were housed in light controlled (14 h light/10 h dark), air-conditioned, specific pathogen free conditions and received food and water *ad libitum*. 1 mg/kg Dex and equimolar amounts of Cortivazol or AZD2906 were gavaged per oral in a volume of 100 μl/20 g bodyweight, prepared in 1% w/v Hydroxypropylmethylcellulose (HPMC), 0.5% v/v Tween 80 and 98.5% v/v distillated water. 50 μg mTNF in a volume of 200 μL sterile PBS/20 g bodyweight was injected intraperitoneally 1 h after the oral gavage. For pharmacokinetics 6–8 weeks old female CD1 mice were used (Shangai SLAC Laboratory Animal Co. LTD, 6–8 weeks old). Mice were orally gavaged with 1 mg/kg Dex, Cortivazol or AZD2906 in a volume of 100 μl/20 g bodyweight, prepared in HPMC solution. All experiments were approved by the institutional ethics committee for animal welfare of the Faculty of Sciences, Ghent University, Belgium. The methods were carried out in accordance with the relevant guidelines and regulations.

### Sample collection and plasma preparation

Blood was sampled from the retro-orbital plexus during isoflurane sedation (Isoflo, Abbot animal health) in EDTA-2K tubes. Blood was maintained on ice for maximally 15 min and then centrifuged (2000 × g, 4 °C, 5 min) to obtain plasma. Plasma was temporally stored on dry ice and transferred to −80 °C for long-term preservation.

### LC-MS

MS was performed at the Metabolomics Expertise Center – CCB, VIB. To 20 µl of plasma, 980 µl of an 80% methanol (80/20 methanol/water, both LC-MS grade) solution was added, the mixture was placed overnight at −80 °C. Next, insolubilities (precipitated proteins) were removed by centrifugation for 15 min at 20.000 × g at 4 °C. The supernatant was dried (vacuum centrifuge) and re-dissolved in 100 µl of 80% methanol. Samples were stored at −80 °C until analysis.

25 µl of the extract was injected using a Vanquish UPLC (Thermo Scientific) equipped with an Acquity UPLC HSS T3 C18 column (dimensions: 2.1 × 100 mm, 1.8 μm particles). Upon injection a linear gradient was carried out using solvent A (0.5% formic acid) and solvent B (100% LC-MS grade methanol) as follows: from 0 to 1 min 10% A, from 1 to 5 min an increase to 95% B was accomplished and maintained until 9 min. From 9 min to 10 min a drop to 50% B was carried out and at 12 min the gradient returned to 10% B. The run stopped at 17 min. The flow was kept constant at 300 μl/min and the column was heated at 40 °C.

For the detection of the compounds the MS (Quantiva triple quadrupole (Thermo Scientific)) operated in MRM mode from 0.1 min to 17 min focusing on the following parent to fragment transitions: Dex (m/z 393.4 → 310.84 at a collision energy of 14.75 V), AZD2906 (m/z 461.2 → 165.2 at a collision energy of 23.35 V) and the Cortivazol metabolite (m/z 489.3 → 459.3 at a collision energy of 37.05 V). The MS operated in positive ion mode, the spray voltage was static (3500 V) and gas settings were as follows: sheath gas at 50, auxiliary gas at 15 V and sweep gas at 2. The ion transfer tube was heated at 350 °C and a vaporizer temperature of 400 °C was applied. A cycle time of 0.4 sec was used and the resolution of the Q1 was 0.7 Da and Q3 1.2 Da. The CID gas operated under a pressure of 1.5 mTorr using Argon as collision gas.

### Statistical analysis

For the luciferase, gene-expression, FRET and ELISA tests a Hierarchical Generalized Linear Mixed Model (HGLMM; fixed model: poisson distribution, log link; random model: gamma distribution, log link) as implemented in Genstat v18^73^ has been fitted to the data. The linear predictor vector of the values can be written as follows:

$${\rm{l}}{\rm{o}}{\rm{g}}(\mu )=\eta ={\bf{X}}\beta +{\bf{Z}}\nu $$

*Χ, β, Ζ and* ν *are defined per experiment in the supplemental methods*. T statistics were used to assess the significance of fixed COMPOUND (and CONCENTRATION effects in case of the luciferase assays) estimated as differences (on the transformed scale in case of the luciferase, RT-qPCR and ELISA tests) to NI, DEX (at a particular concentration in the luciferase assays) or to TNF set as reference. Estimated mean values were obtained as predictions from the HGLMM, formed on the log scale (in case of the luciferase assays) or on the scale of the response variable (in case of RT-qPCR, FRET and ELISA). Survival curves were compared with a Mantel-Cox test in GraphPad Prism 7 (GraphPad Software, San Diego, CA). Plasma PK profiles were compared per timepoint to Dex by means of an unpaired T test in Graphpad Prism 7.

## Electronic supplementary material

Supplementary Dataset 1

## Data Availability

The data generated during and/or analyzed during the current study are available from the corresponding author on reasonable request.
