# Supplementary material for: A screening assay for Selective Dimerizing Glucocorticoid Receptor Agonists and Modulators (SEDIGRAM) that are effective against acute inflammation
Source: Sci Rep. 2018 Aug 27;8:12894. doi: 10.1038/s41598-018-31150-w (PMC6110732; doi:10.1038/s41598-018-31150-w)
Supplement: Supplementary file 1 — Supplementary Dataset 1 [file 41598_2018_31150_MOESM1_ESM.pdf]

A screening assay for Selective Dimerizing Glucocorticoid Receptor Agonists and Modulators (SEDIGRAM) that are effective against acute inflammation

Jolien Souffriau[1,2], Melanie Eggermont[1,2], Sara Van Ryckeghem[1,2], Kelly Van Looveren[1,2], Lise Van Wyngene[1,2], Evelien Van Hamme[3], Marnik Vuylsteke[4], Rudi Beyaert[1,2], Karolien De Bosscher[5,6], Claude Libert[1,2*]

[1] Center for Inflammation Research, VIB, Ghent, Belgium

[2] Department of Biomedical Molecular Biology, Ghent University, Ghent, Belgium

[3] Bio Imaging Core, Center for Inflammation Research, VIB, Ghent, Belgium

[4] GNOMIXX ltd, Statistics for Genomics, Melle, Belgium

[5] Receptor Research Laboratories, Nuclear Receptor Lab, Medical Biotechnology Center, VIB, Ghent, Belgium

[6] Department of Biochemistry, Ghent University, Ghent, Belgium

*Corresponding author:

Claude.Libert@IRC.VIB-UGent.be

Supplemental methods: definition of X, β, Z and ν

$$\log(\mu) = \eta = \mathbf{X}\beta + \mathbf{Z}\nu.$$

Luciferase test:

The matrix **X** is the design matrix for the fixed COMPOUND, CONCENTRATION and the COMPOUND.CONCENTRATION interaction terms, β is the vector of corresponding regression coefficients, **Z** is the design matrix for the two random REPLICATE (representing the biological replicates) and PLATE terms with PLATE nested within REPLICATE, and ν is the corresponding vector of the random effects having a gamma distribution.

Gene expression & ELISA test:

The matrix **X** is the design matrix for the fixed COMPOUND term, β is the vector of corresponding regression coefficients, **Z** is the design matrix for the random REPLICATE term (representing biological replicates), and ν is the corresponding vector of the random REPLICATE effect having a gamma distribution.

FRET test:

The matrix **X** is the design matrix for the fixed COMPOUND term, β is the vector of corresponding regression coefficients, **Z** is the design matrix for the random REPLICATE term (representing biological replicates), and ν is the corresponding vector of the random REPLICATE effect having a normal distribution.

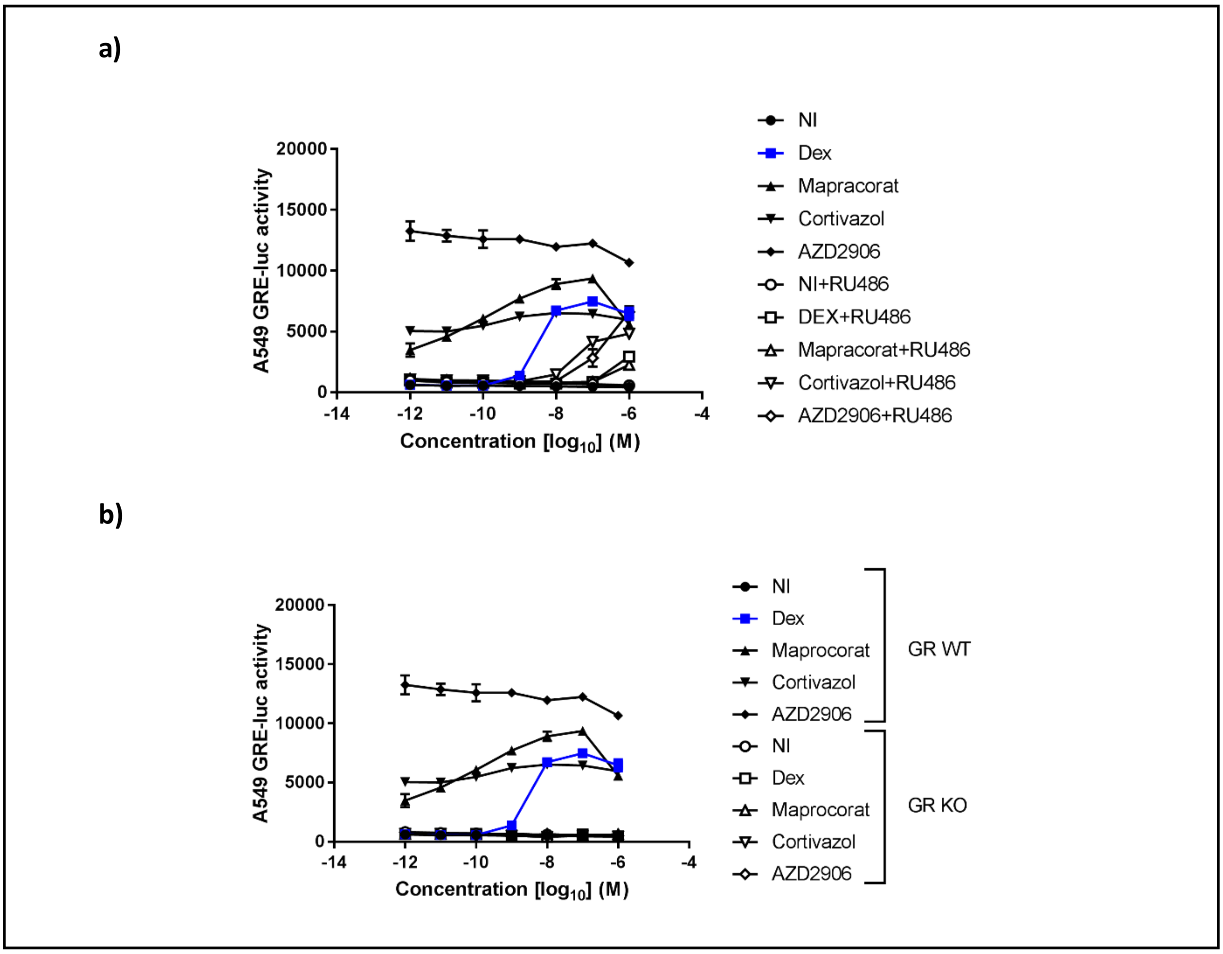

Supplemental Figure S1. The GRE-luciferase signal induced by the compounds is glucocorticoid receptor (GR) dependent. A549 GRE-luc cells were stimulated with a dilution series of compounds for 5 h, after which they were lysed and luminescence was read out. In **(a)** the cells were co-treated with 10^{-6} M RU486. In **(b)** A549 GR KO GRE-luc cells were used. NI: Non-Induced, Dex: Dexamethasone, WT: Wild-Type, KO: Knock-Out, GRE: Glucocorticoid Responsive Element.

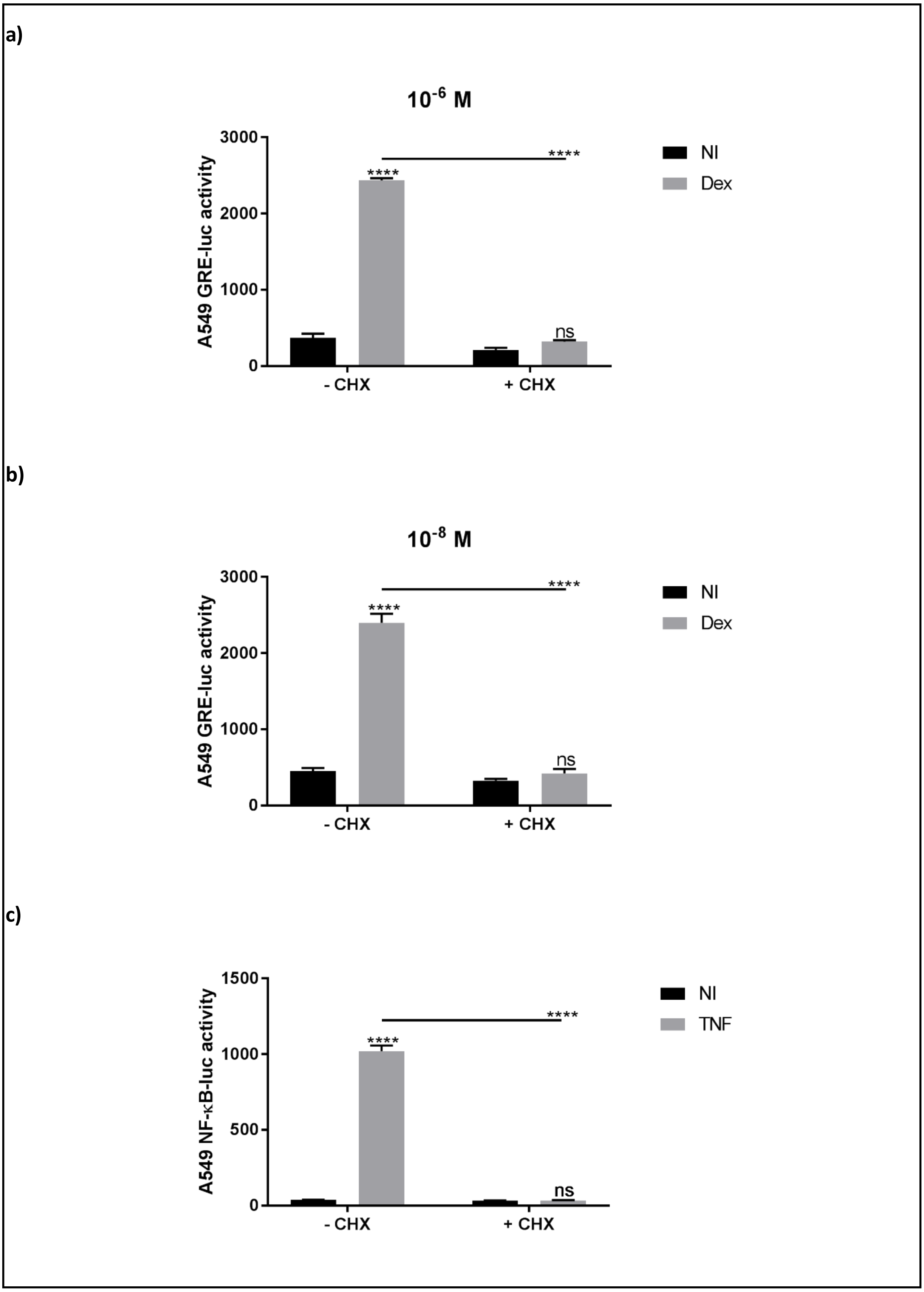

Supplemental Figure S2. Luciferase activity confirms blocking of translation by cycloheximide. A549 GRE-luc and NF-κB-luc cells were pretreated with or without 10 μG/ml cycloheximide (CHX, 30 min). CHX treatment in A549 GRE-luc was followed by a 3 h solvent control (NI) or Dex stimulation at **(a)** 10^{-6} M or **(b)** 10^{-8} M. **(c)** In A549 NF-κB-luc cells CHX treatment was followed by the solvent control for 1h

and subsequently cells were stimulated with 1000 U/ml human TNF for 2 h. The cells were lysed and luminescence was read out. Data are shown as mean ± SEM. Significant differences are calculated with a Two-way Anova. Statistical differences compared to NI (above error bars) or between the Dex or TNF stimuli (indicated with −) are given. **** represents a P-value <0.0001. Ns: Not significant, NI: Non-Induced, Dex: Dexamethasone, TNF: Tumor Necrosis Factor.

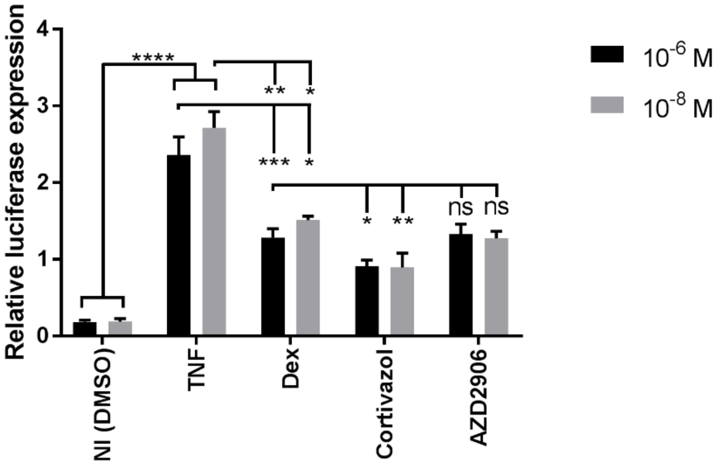

Supplemental Figure S3. Comparison of maximal NF-κB luciferase transrepression over concentrations of GR dimer favouring compounds in absence of novel protein synthesis. NF-κB-luciferase gene-expression in A549 NF-κB-luc cells pretreated with 10 µg/ml cycloheximide (CHX, 30 min), followed by stimulation with the compounds (10^{-6} M and 10^{-8} M). 1 h later cells were challenged with 1000 U/ml human TNF for 2 h. Luciferase mRNA expression was measured with RT-qPCR. Data are shown as mean ± SEM. Significant differences are calculated with a Two-way Anova. *,**,*** or **** represent significant differences with P <0.05, 0.01, 0.001 and 0.0001 respectively. 3 experiments were pooled. Ns: Non-significant, NI: Non-Induced, TNF: Tumor Necrosis Factor, Dex: Dexamethasone.

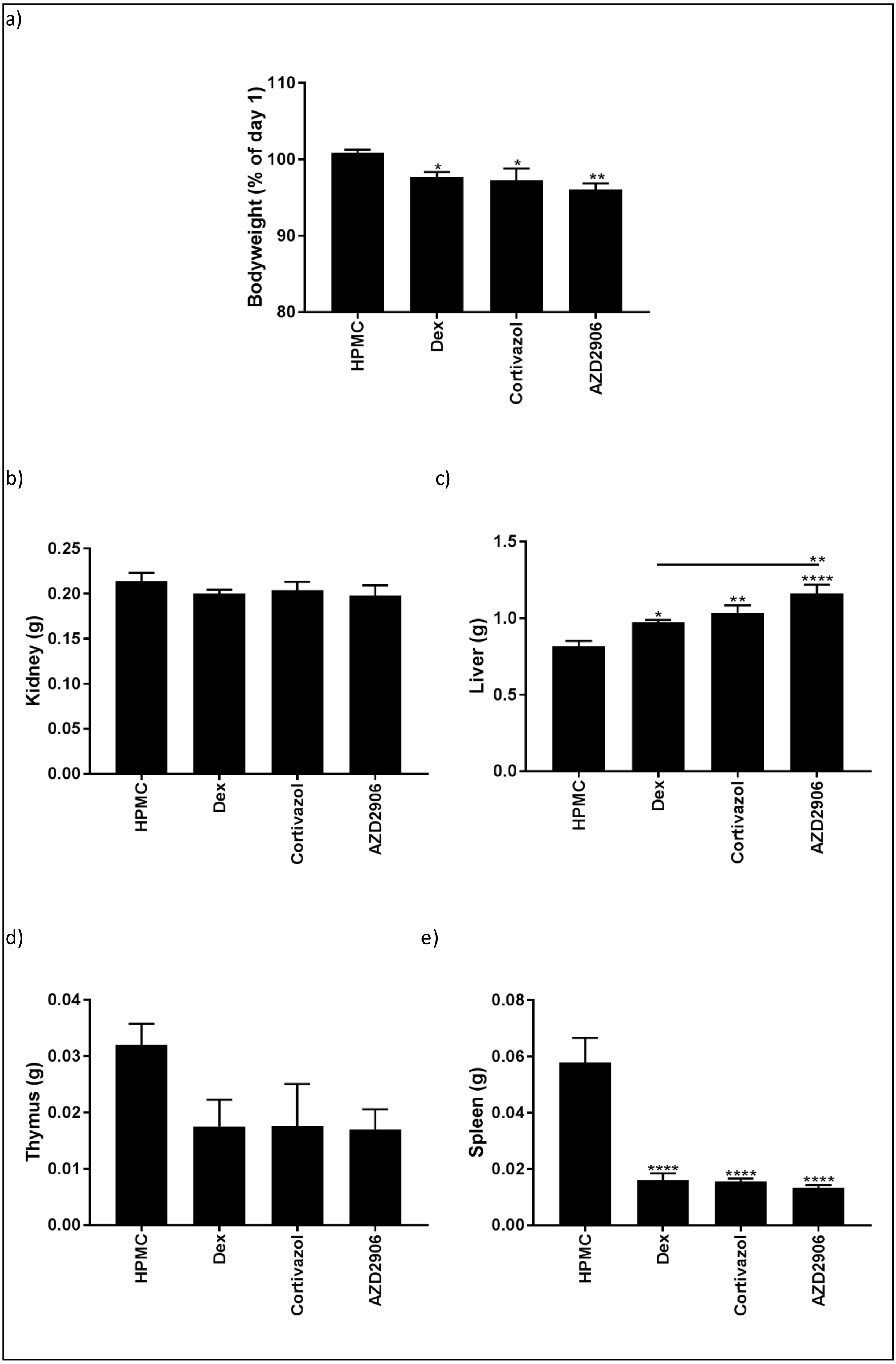
a)
Bodyweight (% of day 1)
110
100
90
80
*
*
**
HPMC
Dex
Cortivazol
AZD2906
b)
Kidney (g)
0.25
0.20
0.15
0.10
0.05
0.00
HPMC
Dex
Cortivazol
AZD2906
c)
Liver (g)
1.5
1.0
0.5
0.0
**
*
**
****
HPMC
Dex
Cortivazol
AZD2906
d)
Thymus (g)
0.04
0.03
0.02
0.01
0.00
HPMC
Dex
Cortivazol
AZD2906
e)
Spleen (g)
0.08
0.06
0.04
0.02
0.00
****
****
****
HPMC
Dex
Cortivazol
AZD2906

Supplemental Figure S4. Dex, Cortivazol and AZD2906 induce glucocorticoid side effects. Female C57BL/6J mice were treated daily with 1 mg/kg Dex or an equimolar dose of Cortivazol or AZD2906 dissolved in 100 µl HPMC, by oral gavage, for 5 consecutive days. **(a)** Whole bodyweight was measured and represented as a percentage of the bodyweight of day 1. **(b)** Kidney, **(c)** liver, **(d)** thymus and **(e)** spleen were isolated and weighed 6 h after the last oral gavage on day 5. Significant differences are calculated with a One-way Anova test. Statistical differences compared to HPMC (above error bars) and Dex (indicated with −) are given. P-values < 0.05, 0.01 and 0.0001 are represented by *, ** or **** respectively. Non-significant differences are not indicated on the graph. N=5 mice per group. HPMC: Hydroxypropylmethylcellulose, Dex: Dexamethasone.

Supplemental Table S5: Statistical significances A549 GRE luciferase test

	10^{-6} M			10^{-7} M			10^{-8} M		
	tvalue	t_Prob		tvalue	t_Prob		tvalue	t_Prob	
NI (DMSO)	-12,0194	0	****	-10.5178	0	****	-10.1074	0	****
Fosdagrocorat	-9.81095	0	****	-7.98583	3.33067E-15	****	-7.1263	1.775E-12	****
Prednisolone	-1.67533	0.094130019	ns	-2.47463	0.013474763	*	-4.9483	8.5516E-07	****
Mapracorat	-1.34209	0.179819598	ns	0.741652	0.458443248	ns	3.1998	0.00141136	**
Cortivazol	-1.50158	0.133467994	ns	-0.31341	0.754021013	ns	0.4723	0.63676508	ns
LGD5552	-7.34966	3.66374E-13	****	-7.0909	2.26996E-12	****	-7.6088	5.5733E-14	****
AZD2906	4.67152	3.32704E-06	****	5.970065	3.11913E-09	****	7.7683	1.6875E-14	****

A Hierarchical Generalized Linear Mixed Model (HGLMM) has been fitted to the luciferase activities measured at various concentrations of the compounds. T statistics (unpaired, two-tailed) were used to assess the significance of fixed compound and concentration effects estimated as differences (on the transformed scale) to Dex at a particular concentration set as reference. Estimated mean values were obtained as predictions from the HGLMM, formed on the log scale. T-values and P-values (t_Prob) for the concentrations 10^{-6} M, 10^{-7} M and 10^{-8} M (concentrations where Dex has its maximal activity) are represented in the table. P <0.05, 0.01, 0.001 and 0.0001 are represented with *,**,*** or **** respectively. Ns: Not significant. Experiments were repeated 6 times with at least 2 technical replicates included.

Supplemental Table S6: Statistical significances A549 NF-κB luciferase test

	10^{-6} M			10^{-7} M			10^{-8} M		
	tvalue	t_Prob		tvalue	t_Prob		tvalue	t_Prob	
TNF	4.093525	5.36968E-05	****	5.061932	6.97285E-07	****	5.31003	2.0399E-07	****
Fosdagrocorat	1.698806	0.09031577	ns	2.534819	0.011720736	*	2.363495	0.0186934	*
Prednisolone	0.390697	0.696278242	ns	0.822702	0.411282717	ns	1.977445	0.04883856	*
Mapracorat	1.008059	0.314178525	ns	0.890265	0.373983976	ns	1.278091	0.20213207	ns
Cortivazol	0.08944	0.928787321	ns	0.398634	0.690425207	ns	0.417441	0.67663249	ns
LGD5552	1.483195	0.138994568	ns	2.525585	0.012027034	*	3.725319	0.00023006	***
AZD2906	-0.10878	0.913445047	ns	-0.00309	0.99753567	ns	-0.07995	0.93632506	ns

A Hierarchical Generalized Linear Mixed Model (HGLMM) has been fitted to the luciferase activities measured at various concentrations of the compounds. T statistics (unpaired, two-tailed) were used to assess the significance of fixed compound and concentration effects estimated as differences (on the transformed scale) to Dex at a particular concentration set as reference. Estimated mean values were obtained as predictions from the HGLMM, formed on the log scale. T-values and P-values (t_Prob) for the concentrations 10^{-6} M, 10^{-7} M and 10^{-8} M (concentrations where Dex has its maximal activity) are represented in the table. P <0.05, 0.01, 0.001 and 0.0001 are represented with *,**,*** or **** respectively. Ns: Not significant. Experiments were repeated twice with at least 2 technical replicates included.
